# The α-Gliadins in Bread Wheat: Effect of Nitrogen Treatment on the Expression of the Major Celiac Disease Immunogenic Complex in Two RNAi Low-Gliadin Lines

**DOI:** 10.3389/fpls.2021.663653

**Published:** 2021-04-29

**Authors:** Susana Sánchez-León, María José Giménez, Francisco Barro

**Affiliations:** Department of Plant Breeding, Institute for Sustainable Agriculture – Spanish National Research Council, Córdoba, Spain

**Keywords:** α-gliadins, nitrogen, RNAi wheat, celiac disease, endosperm-specific promoters, gluten, 33-mer

## Abstract

Celiac Disease (CD) is an autoimmune disorder that affects approximately 1% of the worldwide population. The α-gliadins of wheat contain the 33-mer peptide, the most active peptide in CD both in adults and pediatric patients. In this study, we have characterized the variants and expression profile of an α-gliadins amplicon, harboring the 33-mer peptide, in two low-gliadin RNAi wheat lines, under two different Nitrogen (N) treatments. We estimated that the amplicon expands 45 different α-gliadin variants with high variability due to length, randomly distributed SNPs, and the presence of encoded CD epitopes. Expression of this amplicon is reduced in both RNAi lines in comparison to the wild type. High N treatment significantly increases transcripts of the amplicon in the wild type, but not in the transgenic lines. Classification of α-gliadin variants, considering the number of epitopes, revealed that amplicon variants containing the full complement of 33-mer peptide were affected by N treatment, increasing their expression when N was increased. Line D793 provided higher and more stable silencing through different N fertilization regimes, expressing fewer CD epitopes than D783. Results of this study are important for better understanding of RNAi α-gliadin silencing in response to N treatments, and for undertaking new strategies by RNAi or CRISPR/Cas toward obtaining new varieties suitable for people suffering gluten intolerances.

## Introduction

Celiac disease (CD) is an autoimmune disorder triggered by the ingestion of gluten from wheat, rye, or barley in individuals with a genetic predisposition. Although the CD has a global distribution, its prevalence varies greatly depending on the country or region, ranging from 1 to 2% in Western countries (Singh et al., 2018). Individuals with a genetic predisposition for CD carry the class II human leukocyte antigen (HLA)-DQ2 and/or (HLA)-DQ8. The strongest association has been found with HLA-DQ2, being present in at least 90% of the celiac population. The presence of epitopes recognized by HLA-DQ2/DQ8 is a consequence of the incomplete degradation of gluten proteins by intestinal enzymes, resulting in highly stimulatory gluten peptides, varying in length in the intestinal lumen. These peptides are translocated to the lamina propria. An issue that substantially increases the binding capacity of HLA-DQ2/DQ8 molecules is the specific deamidation of glutamine residues to glutamic acid by the type 2 tissue transglutaminase enzyme (tTG2), causing an increase in negative charges in the gluten-derived peptides (Ráki et al., 2007).

Gluten peptides are not derived from a single protein, but from a complex mix of proteins that comprise about 70% of the total protein in wheat grain and can be classified into two major groups: gliadins and glutenins. Gliadins remain mainly as monomers in the grain while glutenins form large aggregates. Gliadins are responsible for the extensibility of wheat doughs, while glutenins are for elasticity (Shewry et al., 2003). Gliadins are divided into three complex fractions, named α-, γ-, and ω-gliadins, which have a similar structure, characterized by the presence of a repetitive region of variable length depending on the fraction. Although gliadins contain cysteine residues, they mainly form intramolecular disulfide bonds. Glutenins are divided into high molecular weight (HMW) and low molecular weight (LMW) glutenin subunits (GS), which form large polymers linked by intermolecular disulfide bonds (Shewry and Halford, 2002).

However, not all gluten proteins are equally important for CD. Experiments with T-cell panels have determined that the adaptive response is restricted to certain gluten peptides that mostly map in gliadin regions (Arentz-Hansen et al., 2002). A comprehensive review of CD-related epitopes identified in wheat, rye, barley, and oats has recently been published (Sollid et al., 2020). Although T-cell reactivity appears to be heterogeneous, response to α-gliadins predominates. In fact, a peptide present in α-gliadins, named 33-mer, induces specific immune responses in almost all patients with CD (Arentz-Hansen et al., 2000; Tye-Din et al., 2010; Hardy et al., 2015). The high immune response of the 33-mer peptide is due to the presence of six overlapping copies of three different DQ2-restricted T-cell epitopes (Shan et al., 2002). The full immunodominant 33-mer fragment—harboring the six epitopes copies—is only present in hexaploid wheat, as the result of allohexaploidization events from other α-gliadins found in *Aegilops tauschii*, the D-genome donor of hexaploid wheat (Ozuna et al., 2015). However, shorter variants of this peptide are also present in diploid, tetraploid, and hexaploid wheat. In addition, flanking the 33-mer peptide, the wheat α-gliadins also contains two CD immunogenic peptides: the p31–43 peptide, which was reported to induce the innate immune response necessary to initiate the T-cell adaptive response (Maiuri et al., 2003); and an additional DQ2-restricted epitope (DQ2.5-glia-α3) (Vader et al., 2002) which partially overlaps with 33-mer peptide. All these three peptides are located in the N-terminal region of the α-gliadins, and they can be expanded by targeted amplicon sequencing, showing enormous genetic variability among wheat cultivars for the abundance in these three peptides (Ozuna et al., 2015).

The application of biotechnological techniques such as RNAi and CRISPR/Cas provided new wheat varieties with immunogenic gliadins strongly down-regulated (Barro et al., 2016; Sánchez-León et al., 2018) showing that these techniques are appealing alternatives for the development of gluten-free cereal varieties that can be tolerated by celiacs or patients suffering other gluten-related pathologies (Haro et al., 2018; Camerlengo et al., 2020). For RNAi varieties, the decrease in the content of α-gliadins was up to 90% (Pistón et al., 2013). Furthermore, these RNAi varieties showed decreases of up to 100-fold in the ability to stimulate the immune response of T cells that specifically recognize α-gliadins epitopes (Gil-Humanes et al., 2010) and an impaired capacity to stimulate Peripheral Blood Mononuclear Cells (PBMCs) from CD patients, providing a stimulatory response that does not differ from the gluten-free negative control (Sánchez-León et al., 2019). In addition, N fertilization is a common practice to regulate yields and protein content in wheat (Nakano and Morita, 2009; Yu et al., 2018), and an increase in N fertilization rates promotes the increment of total gluten content, including all gliadin fractions. These RNAi varieties also showed markedly different responses in relation to N fertilization, related to the RNAi construct used for gliadin silencing, increasing the gliadin content when nitrogen is increased (García-Molina and Barro, 2017).

The objectives of this work were to characterize the amplicon of α-gliadins containing highly stimulatory CD-epitopes in two RNAi lines of wheat with the gliadins strongly down-regulated and to determine if the entire set of genes encompassing this amplicon is equally affected by N fertilization. The results will be of great importance for new innovative RNAi or CRISPR/Cas designs toward obtaining non-immunogenic wheat varieties and to understand how N fertilization affects α-gliadins, allowing low levels of these highly immunogenic proteins in the field.

## Materials and Methods

### Plant Material

Two transgenic lines of the *Triticum aestivum* cv Bobwhite (named BW208), and the wild type were used in this work. Both transgenic lines, D783 and D793, were reported previously (Gil-Humanes et al., 2010) as RNAi lines with all gliadins strongly down-regulated. Lines D783 and D793 were transformed, respectively, with plasmids pDhp_ω/α and pGhp_ω/α, containing the same RNAi fragment, designed to silence all three gliadins groups, but in plasmid pDhp_ω/α (Line D783) the expression of the RNAi fragment was driven by a D-hordein promoter (Pistón et al., 2008) and in plasmid pGhp_ω/α (Line D793) by a gamma gliadin promoter (Pistón et al., 2009).

### Plant Growth and N Treatments

The plant material studied in this works comes from a greenhouse experiment previously described by García-Molina and Barro (2017) (Denoted as “Experiment 2” in the cited publication), where lines D793, D783, and BW208 were grown under two N fertilization levels. Briefly, the trial was carried out as a randomized complete block design, where two replicates of four plants per line and treatment were used. Two nitrogen levels were used considering a threshold fertilization level of about 200 kg N/ha, thus amounts above and below this value were used: low nitrogen (Low N), which represents 120 mg N in total per 1 L pot, and high nitrogen (High N), which represents 1,080 mg N in total per 1 L pot. A basal N fertilization of 60 mg was supplied to both treatment levels at the beginning of the experiment and, to complete the aforementioned dose, an adequate amount of N was applied to each treatment level during the stem elongation stage.

### DNA and RNA Isolation

Young leaves tissue was harvested, grounded in liquid nitrogen, and stored at −80°C until DNA isolation. Genomic DNA was isolated using the CTAB method (Murray and Thompson, 1980) with minor modifications. Six seeds of the central part of the same spike from each plant were collected at 10-, 18-, and 26-days post anthesis (DPA) for total RNA isolation, and immediately frozen in liquid nitrogen and stored in −80°C until RNA extraction. Seeds from the same treatment, developmental stage, and block were bulked, and total RNA was isolated by a TRIzol^®^ Reagent (Invitrogen, Carlsbad, CA) based two-step method for DNA-free RNA extraction (Meng and Feldman, 2010). RNA integrity was verified on 2% agarose gel electrophoresis. RNA and DNA quantity and purity were determined using a NanoDrop ND-1000 spectrophotometer (Thermo Fisher Scientific, Waltham, MA, United States).

### RT-qPCR

All RNA samples were adjusted to the same concentration for subsequent reverse-transcription reactions. cDNA was obtained by reverse transcription from 1 μg of total RNA using iScript^TM^ cDNA Synthesis Kit (BioRad, Hercules, CA, United States) in 20 μl of total reaction volume according to the manufacturer’s instructions. Two cDNA replicates of each sample were pooled.

To quantify relative expression level the most important items of the MIQE checklist (Bustin et al., 2009) were considered as described in a practical approach to RT-qPCR experiments (Taylor et al., 2010). All RT-qPCR reactions were performed using SYBR^®^ Green as fluorophore on a CFX Connect^TM^ Real-Time PCR Detection System (BioRad, Hercules, CA, United States). The qPCR run was performed as a 2-step plus melting curve protocol with an initial denaturation step at 95°C for 3 min followed by 40 cycles of annealing and extension at 61°C for 30 s and denaturation at 95°C for 10 s. For each qPCR reaction, 6 μl of 1:20 diluted cDNA solution was used in 25 μl total volume reaction, containing 12,5 of PerfeCTa^®^ SYBR^®^ Green FastMix^®^ (Quanta Biociences) and 225 nM of each primer. For amplification of the α-gliadin target amplicon the forward primer aGLI900F1 (5′-GTTAGAGTTCCAGTGCCACAA) and reverse primer 33mer1R2_Ok (5′-GGTTGTTGTGGTTGCGRATA) were used. Primers for three housekeeping genes, cell division control protein (CDC), ADP-ribosylation factor (ADP-RF), and RNase L inhibitor-like protein (RLI) (Giménez et al., 2011) were used for comparing the relative expression of the target α-gliadin amplicon. Each qPCR run was performed in triplicate, including no-template controls (NTC) and RNAs as a template to discard DNA contamination. The specificity of the amplifications was confirmed by the presence of a single band of the expected size for each primer pair in agarose gel electrophoresis and by single-peak melting curves profiles of the PCR products.

PCR efficiency of each primer pair was determined by the open-access software application LinRegPCR V12.17 (Ruijter et al., 2009; Tuomi et al., 2010) using raw fluorescence as input data. Expression of the genes (N_0_) was determined for each sample using the equation N_0_ = threshold/E^Cq^, where E = PCR efficiency for each primer and Cq (cycle of quantification) is the number of cycles needed to reach a threshold of arbitrary units of fluorescence. The stability of the reference genes across the sample sets was analyzed using geNorm (Vandesompele et al., 2002) as described in its manual. A normalization factor (NF) for the α-gliadin amplicon was computed by the geNorm program based on the geometric mean of the expression levels of stable reference genes.

### PCR Amplification of Complete α-Gliadin Gene and Sequencing by Sanger

We sequenced 197 clones of full-length α-gliadin genes from the BW208 wheat cultivar. For PCR conditions, the full-length DNA sequences were amplified using primers VH_aGli_F1 (5′-ATGAAGACCTTTCTCATCC-3′) and VH_aGli_R3 (5′-GTTGGTACCGAAGATGCC-3′) and ligated into pGEM-T Easy vector (Promega, Madison, WI, United States). Full-length genes were cloned into *Escherichia coli* DH5α cells and sequenced by the STABVIDA sequencing service (Caparica, Portugal). Geneious prime version 2020.1.1 from Biomatters Ltd. (Auckland, New Zealand^1^) was used to assemble the sequences from the Sanger sequencing. Sanger sequences were deposited in GenBank under the Accession Nos. MW464662 to MW464858.

### Amplicon Sequencing by Illumina Technology

Forty samples were subjected to Illumina sequencing of the α-gliadin amplicon: four samples corresponded to BW208 DNA, and 36 samples to cDNA from lines D793, D783, and BW208, which correspond to low and high N levels at 10-, 18-, and 26-days post anthesis (DPA). The α-gliadin amplicon was sequenced by the Illumina MiSeq system producing 2 × 300 paired-end reads. Illumina amplicon library was prepared and sequenced at Unidad de Genómica of Fundación Parque Científico de Madrid (FPCM, Madrid, Spain) as previously reported (Sánchez-León et al., 2018) with some modifications. Briefly, to avoid the bias of the sequencing results, a dilution of all samples considering the Cq was made to start the sequencing with the same amount of α-gliadin amplicon molecules, and first PCR was terminated at the exponential phase to maintain the proportionality of amplicon variants. The range of amplicon lengths was check using the Agilent 2100 Bioanalyzer system (Agilent Technologies, Santa Clara, CA 95051). The raw datasets generated can be found at NCBI sequence read archive under Accession No. SRR13281228 to SRR13281247.

### Gene Cluster Analysis

In total, 4,032 million reads were obtained, which correspond to an average of 100,000 reads per sample. For clustering, the USEARCH software v9.2.64 (Edgar, 2010) was used. Reads were preprocessed and filtered using the -fastq_mergepairs and -fastq_filter commands, respectively, with a minimum amplicon length of 160 bp (-fastq_minmergelen 160), and quality filtering by expected errors (-fastq_maxee 1) (Edgar and Flyvbjerg, 2015). Then, filtered reads were used to recover biological sequences using the UNOISE algorithm for error-correction and chimera detection (Edgar and Flyvbjerg, 2015). Search and clustering of reads were carried out at 99% homology. To extract the high-confidence amplicon variants for each sample, the amplicons supported by at least 75 reads in at least 3 samples were kept. Reads were normalized to the sample with the lowest number of reads and considering the dilution factor and RT-qPCR NF.

### BW208 α-Gliadin Amplicon Database

All α-gliadin sequences from Sanger and Illumina were processed to determine the number of amplicons and to construct the BW208 amplicon database, which was Blastn against the NCBI α-gliadins sequences. Then, the resulting BW208 amplicon database was used for clustering of all cDNA reads.

### Statistics

Statistical software R version 3.5.1 (R Development Core Team, 2010) was used to perform the data analysis and some of the graphs drawing. Analysis of variance (ANOVA) followed by the two-tailed Dunnett test for mean multiple comparisons was used for establishing differences between lines or treatments. Normal distribution and homogeneity of variance were previously tested by the Shapiro–Wilk normality test and the Levene test, respectively. Figures were drawn using the Microsoft Excel and PowerPoint software (Microsoft Corporation) and R software. Library ComPlexHeatmap Heat map was used to construct the heatmap (Gu et al., 2016). Principal Component Analysis (PCA) of aggregated data (sum of the three DPA) of amplicon abundance adjusted for α-gliadin expression for each genotype and N fertilization level was performed. The values of the contents of grain prolamin and its fractions at harvest, previously reported by García-Molina and Barro (2017), were included as Supplementary Data in the PCA. The libraries FactoMineR (Lê et al., 2008) and Factoextra were used for PCA analysis and graphical output, respectively.

## Results

**FIGURE 1:**
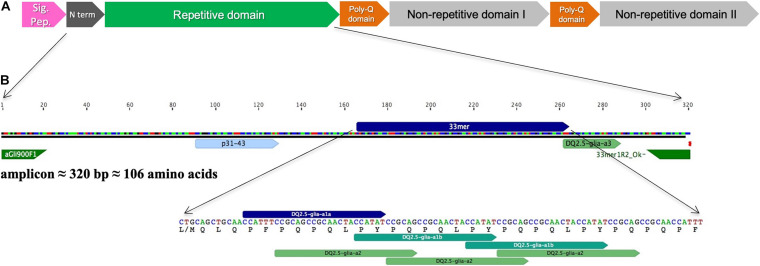
**(A)** Schematic representation of full-length α-gliadin gene (Accession No. AJ133602) as reviewed in Qi et al. (2006) indicating the protein domains. **(B)** Amplicon fragment and the main immunogenic region in which the peptides p31–43, 33-mer and DQ2.5-glia-a3, and primers used to amplify the amplicon are indicated.

In this work, we have sequenced variants of an amplicon from wheat α-gliadin genes that contain highly stimulatory CD-epitopes (Figure 1) and characterized their expression in two RNAi lines with the gliadin genes strongly down-regulated. Lines were subjected to two different N fertilization regimes and the expression profile of the amplicon variants was evaluated at three developmental stages of the grain.

### DNA-Amplicon Variants

**FIGURE 2:**
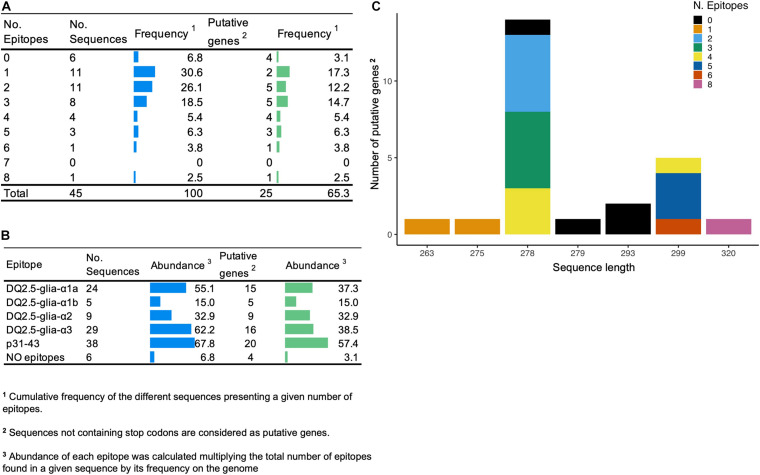
**(A)** Number of epitopes and frequency of the amplicon variants. **(B)** Abundance of DQ2.5 restricted epitopes and p31-43 non-immunodominant peptide in amplicon variants. **(C)** Distribution of the different putative genes that comprise the amplicon in terms of length and number of epitopes.

First, the number of high-confidence amplicon variants was determined using DNA from the BW208 wild type by Illumina Next Generation Sequencing (NGS) and Sanger sequencing. To gather the high-confidence α-gliadin variants, amplicons supported with less than 75 Illumina reads were discarded. As showed in Supplementary Figure 1, 45 different amplicon variants were found in this wheat background, of which 20 were pseudogenes as they present stops codons (Figure 2). The complete list of amplicons and their characteristics can be found in Supplementary Table 1. Amplicons were addressed to the A, B, and D genome as described by van Herpen et al. (2006). Amplicon sequences were then searched for CD-relevant gluten epitopes recognized by CD4^+^ T cells (Sollid et al., 2020), and also for the non-immunodominant peptide p31-43 associated with the innate response to gliadins (Maiuri et al., 2003). The two major variants of the peptide p31-43 (LG-, and LP- type) were considered (Ozuna et al., 2015). DNA amplicon sequences containing 1–3 CD epitopes were the most abundant. Notably, six amplicon variants (four putative genes) do not contain CD-related epitopes, although their overall abundance is low (Figure 2A). The most abundant amplicon (Amp19, genome B) does not contain any 33-mer neither DQ2.5-glia-α3 epitopes but the p31-43 peptide (Supplementary Table 1). The p31-43 peptide is the most abundant, presents in 38 out of 45 amplicons—and in 20 of the 25 putative genes (Figure 2B). Of the three epitopes that make up the 33-mer peptide, DQ2.5-glia-α1a is the most abundant and presents in a higher number of sequences. Figure 2C shows the length of the amplicons and the number of epitopes per amplicon considering only the putative genes.

### Analysis of RNAi Silencing Fragment and α-Gliadin Gene Family Expression by RT-qPCR

**FIGURE 3:**
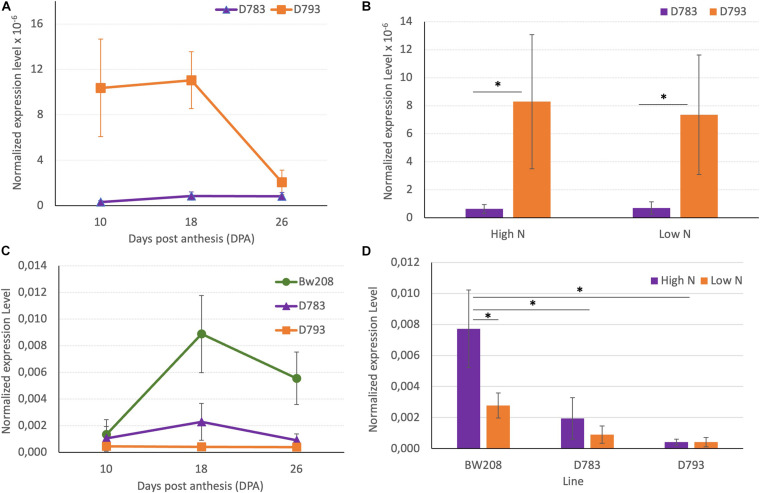
**(A,B)** Normalized expression of RNAi silencing fragment. **(C,D)** Normalized expression level of α-gliadin amplicon. The asterisk (^∗^) above horizontal lines indicates significant difference (*P* ≤ 0.05) between treatments according to the variance analysis. Vertical bars represent the standard deviation.

Lines D783 and D793 share the same RNAi silencing fragment, and the main difference between them is the promoter that directs the expression of this silencing fragment (Gil-Humanes et al., 2010). Although in both lines the down-regulation of gliadins was highly effective, they showed differences in grain protein accumulation in response to N fertilization (García-Molina and Barro, 2017). Figures 3A,B show the expression of the RNAi silencing fragment determined by means of RT-qPCR during grain development, and at the two N treatments (low and high) tested in this study. Expression of the RNAi silencing fragment was significantly higher for line D793 at 10- and 18-days post anthesis (DPA) than that of line D783 (Figure 3A). Expression of this RNAi fragment was not affected by N treatment in either line, although there are significant differences between the two lines, both at low N and high N (Figure 3B).

Then, we used RT-qPCR to determine the expression of the α-gliadin gene family in the BW208 wildtype line and the two RNAi-derived lines. At 18 and 26 DPA, the expression level of the α-gliadins in the wildtype was higher than that of the RNAi lines, while no significant differences between the two RNAi lines were found (Figure 3C). N fertilization has a significant clear effect on the expression of the α-gliadins in the BW208 wildtype, increasing the expression when increasing nitrogen. Although no statistically significant differences were detected between high and low N for both RNAi lines (Figure 3D), there is a trend to increase the expression of the α-gliadins amplicon in line D783 when N is applied, while line D793 appears to be more stable in both N treatments.

### Abundance of α-Gliadin Variants Transcripts

**FIGURE 4:**
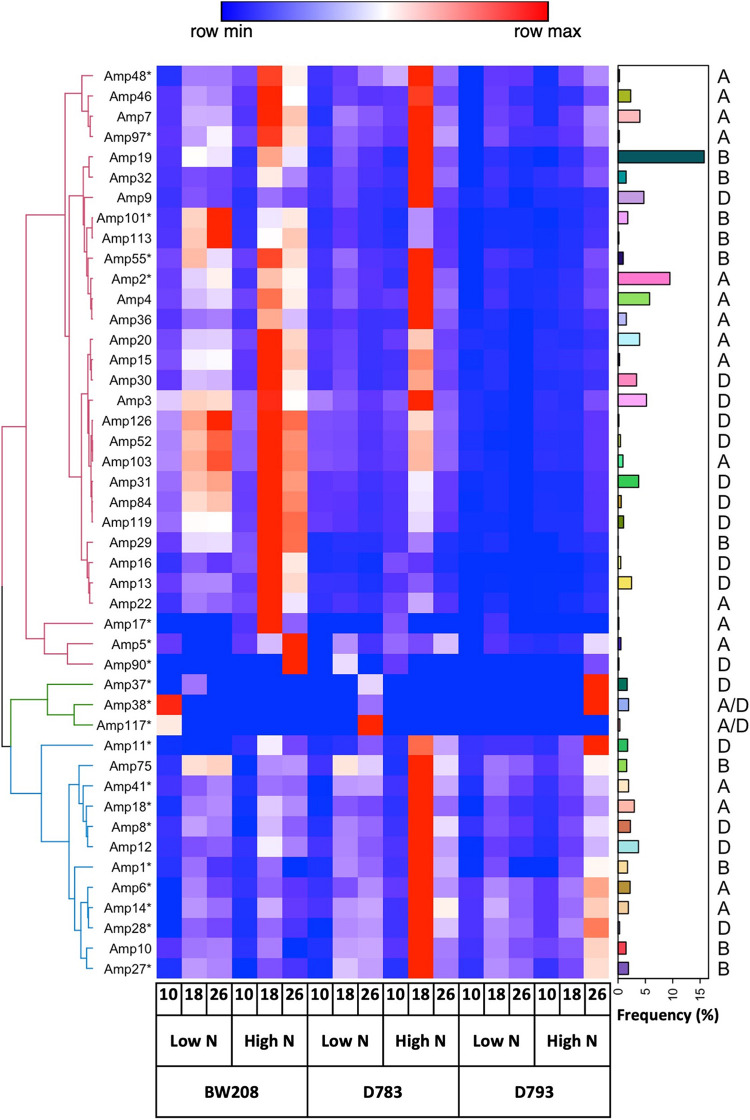
Clustered heatmap in a divergent scale of normalized cDNA reads from the wild type (BW208) and RNAi (D783 and D793) lines at 10-, 18-, and 26-days post anthesis, and at low and high nitrogen (N). Amplicon variants with asterisks are pseudogenes. Amplicon variants were clustered by hierarchical clustering—spearman correlation method. Bar plot indicates the DNA frequency of amplicon variants. The corresponding hexaploid wheat genome is also indicated.

An amplicon database resulting from DNA sequencing was used for clustering cDNA-amplicons from each genotype, N treatment, and grain development stage (date post-anthesis). We characterized the expression pattern of all 45 amplicon variants and represented it in a heat map for the three stages of grain development at low and high N (Figure 4). To facilitate monitoring, we also plotted the frequency (%) of each of the α-gliadin variants in the DNA and their corresponding hexaploid wheat genome. Hierarchical clustering showed an upper group of clusters mainly composed of putative genes, and a lower cluster comprising most of the pseudogenes. For the BW208 wildtype line, the highest change between high and low N occurs between 18- and 26-DPA, involving the group of clusters formed by putative genes, while the cluster comprising pseudogenes is not affected by N. The two RNAi lines showed similarities and differences between the high and low N treatments. While for the D783 line almost all amplicon variants (genes and pseudogenes) showed high changes at 18-DPA, for the D793 line the highest changes occur at 26-DPA and only for clusters containing pseudogenes. The most nitrogen responding amplicon variants were addressed to the A and D genome.

We wanted to learn differences between the three wheat genomes, both for the distribution of CD epitopes and for the expression of α-gliadin amplicons, considering only the putative genes (Supplementary Figure 2). The number of amplicons is higher for the D genome, also its abundance. It should be noted that of the 6 amplicons addressed to genome B, none contain 33-mer epitopes, only p31-43 and DQ2.5-glia-α3 in two amplicons. In contrast, the amplicons assigned to genome D contain epitopes of the 33-mer, also the DQ2.5-glia-α3 epitope, and the p31-43 peptide. Amplicon expression data were aggregated for each of the three wheat genomes, genotypes, and N treatments, and the expression ratio between high and low nitrogen calculated (Supplementary Figure 2). Aggregated expression was notably increased at high nitrogen for the α-gliadin amplicons from A and D genomes, particularly for line D783. The fold change for this line was fourfold at high nitrogen, irrespective of the genome (Supplementary Figure 2, Inset). Lines BW208 (wildtype) and D793 showed comparable ratios, but the RNAi fragment of line D793 was highly effective in the down-regulation α-gliadin amplicons from all three genomes.

**FIGURE 5:**
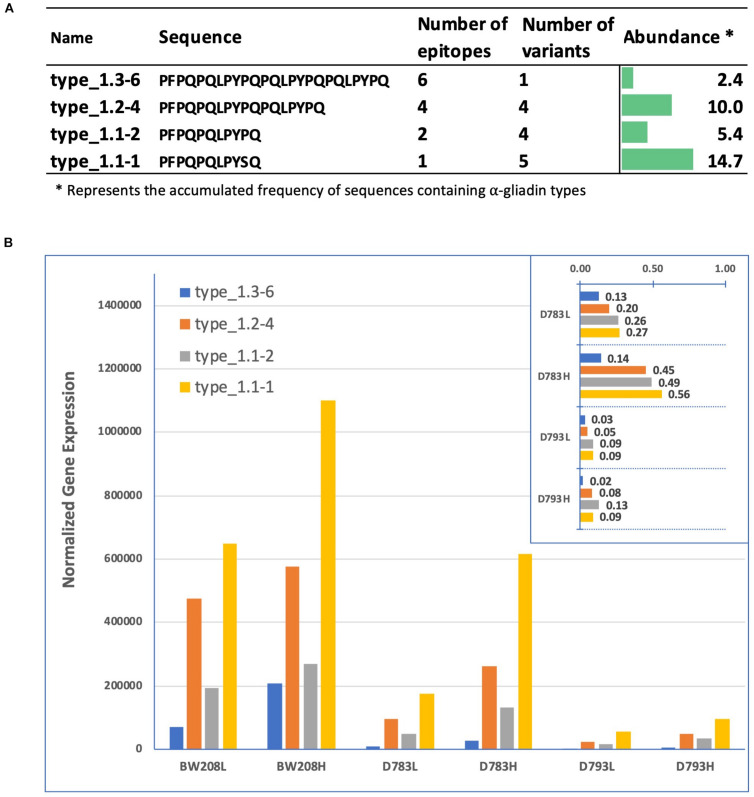
**(A)** Type 1 α-gliadin sequences, number of copies of the 33-mer epitopes, number of amplicon variants containing them, and abundance in the genome. Only putative genes were considered. **(B)** Normalized gene expression of the type 1 α-gliadins in the three genotypes and Nitrogen treatments. Inset; expression ratio of D783 and D793 in comparison to that of the wild type BW208. Letters “L” and “H” following the name of the wheat lines mean Low nitrogen and High nitrogen, respectively. ^∗^, abundance was calculated multiplying the total number of amplicons containing type 1 sequences by its frequency in the genome.

We have previously identified six types of α-gliadins (named 1–6) with strong differences in their frequencies in diploid and polyploid wheat and in the presence and abundance of CD immunogenic peptides (Ozuna et al., 2015). Notably, only type 1 α-gliadins contained 33-mer epitopes, with several subtypes according to the number of copies of the 33-mer epitopes. We searched the type 1 sequences in amplicon putative gene variants and quantified their expression for each genotype under low (L) and high (H) N treatment (Figure 5). All types of α-gliadins were found in amplicon variants addressed to the D genome. The full set of six epitopes comprising the 33-mer are present only in one amplicon (Amp13), which represent 2.4% of the total α-gliadin amplicons in the genome. Variants with higher frequency comprise those containing 1 and 4 copies of the CD epitopes, present in 5 and 4 sequences, respectively (Figure 5A). The overall expression of the 4 α-gliadin types is increased at high nitrogen, and particularly type 1.1-1, with only one epitope, for lines BW208 and D783. Remarkably, line D793 showed the lowest expression levels for all α-gliadin types. The effectiveness of the RNAi silencing fragment becomes apparent when we normalize the expression levels of the D783 and D793 lines at low and high N with respect to that of the BW208 (Figure 5, inset). Line D793 is effective in maintaining very low expression levels for the 4 types of gliadins, while line D783 is not.

### PCA Analysis

**FIGURE 6:**
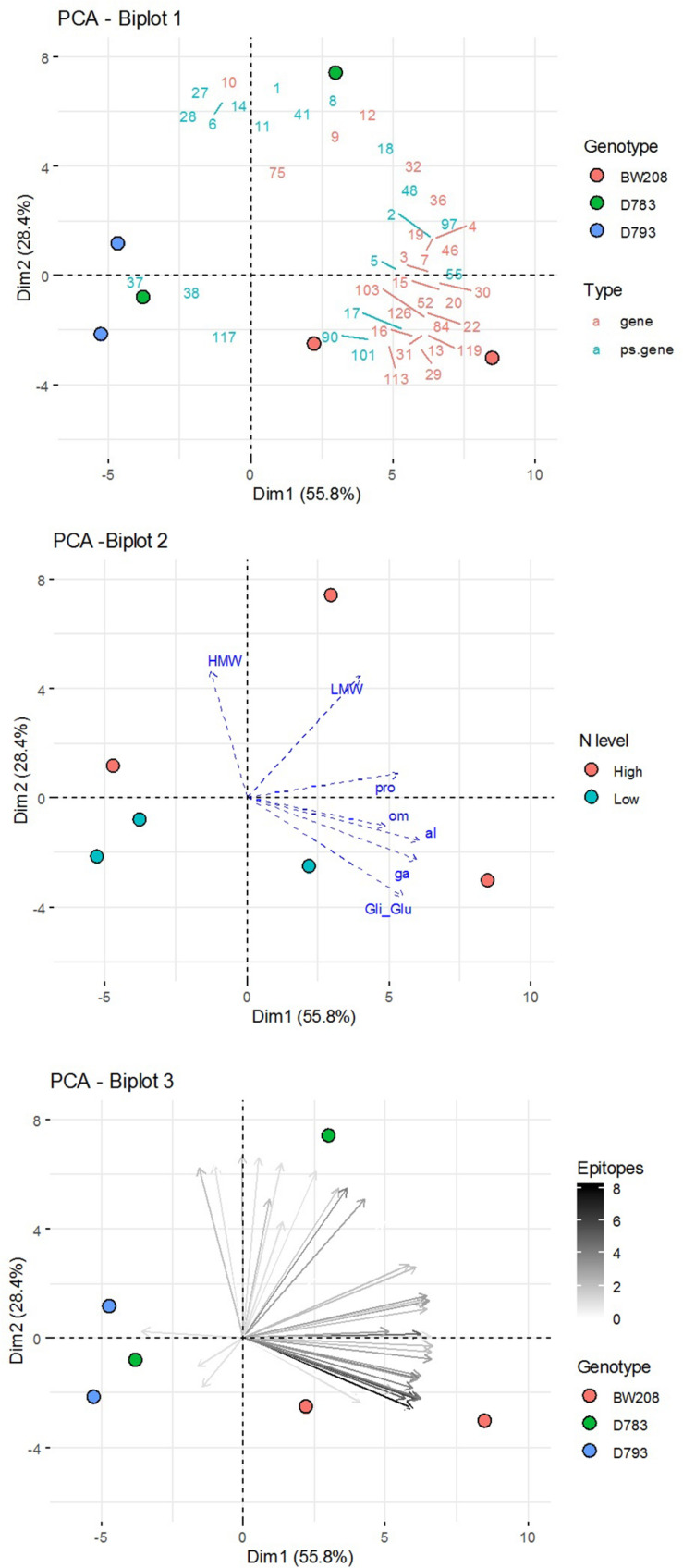
Principal Components Analysis (PCA) of aggregated data (sum of the three DPA) of amplicon abundance for each genotype and N fertilization level. Biplots of: (1) variables grouped by Type (genes or pseudogenes) and groups of individuals (Genotypes); (2) individuals grouped by N fertilization level and supplementary variables (contents of grain prolamin and its fractions); and (3) variables (amplicons) in a color gradient according to the number of epitopes, and individuals (Genotypes).

Finally, a Principal Components Analysis (PCA) of transcripts abundance of each α-gliadin variant for each genotype and N fertilization level was carried out (Figure 6). The total α-gliadin content of the grain is the product of the translation of each of the α-gliadin genes expressed throughout its filling. We have estimated the total expression by aggregating each of the amplicons to perform a PCA analysis that allows us to figure out the relationships between them. Representation of the genotypes on PCA axis 1 and 2—explaining more than 80% of the observed variation (Figure 6) shows that the wildtype genotype is associated with increased cumulative gene expression, particularly those with higher epitopes, both at high and low N levels. In contrast, RNAi genotypes are inversely related or unrelated to the increase in the amounts of these genes.

The grain gliadin contents, previously reported by García-Molina and Barro (2017), have also been represented as supplementary variables over these PCA axes, showing that the variation in the content of gliadins, especially α- and γ-gliadins, is related to a differential accumulated expression of certain α-gliadin variants, most of them corresponding to putative genes and containing the highest number of epitopes.

## Discussion

Although gluten proteins are important for the bread-making quality of wheat, they are also responsible for triggering CD in genetically predisposed individuals. However, not all gluten proteins are equally immunogenic for CD, being the α-gliadins responsible for most of the immune response after eating wheat-containing foods (Tye-Din et al., 2010). The 33-mer peptide, containing six overlapping copies of three CD epitopes, is the main immunogenic peptide found in the α-gliadins. However, this peptide contains many variants that differ in length, in the number of CD epitopes, and in their abundance and reactivity in wheat cultivars (Ozuna et al., 2015; Ruiz-Carnicer et al., 2019). A better understanding of the CD epitopes composition and transcriptional regulation and silencing of the different α-gliadins gene variants is of significant interest in order to develop new RNAi or CRISPR/Cas designs toward obtaining non-immunogenic wheat varieties with minor impact on flour quality. In this work, we report the variants and pattern of expression of a target amplicon of α-gliadins containing highly immunogenic CD epitopes, including the 33-mer peptide, in two RNAi lines with gliadin genes strongly down-regulated under two N treatments.

We have found 45 different amplicon variants sequencing the BW208 wild type by Sanger and Illumina technology. This number of amplicon variants is comparable to the 47 α-gliadin genes annotated in the reference genome of bread wheat variety “Chinese Spring” recently released by the International Wheat Genome Sequencing Consortium (Huo et al., 2018). Furthermore, 45% of the variants were identified as pseudogenes, which is in accordance with the high rate of pseudogenes associated with the dynamic sequence evolution of prolamins (Huo et al., 2018). Even so, the pseudogene rate may be underestimated as, due to size limitation, the amplicon does not cover the complete α-gliadin gene (Figure 1).

Variants analysis revealed high variability in terms of length and epitope abundance, where variants with the higher number of CD epitopes were identified in the D genome, as previously reported (Ozuna et al., 2015; Wang et al., 2017). Sequencing results also agree on the lowest abundance of epitopes in the α-gliadin genes of the B and A genomes, respectively. Tye-Din et al. (2010) found that in wheat, the highly stimulatory 33-mer peptide could elicit most of the immune response observed in CD patients. This peptide is associated with the D genome of hexaploid wheat, explaining the higher immunogenic potential of bread wheat, however, the complete 33-mer peptide is found at a low frequency of 2,47% in this particular genotype. In addition, four of the putative α-gliadin variants do not contain any CD-related epitopes. These findings agree with previous works (Molberg et al., 2005; van Herpen et al., 2006) evidencing that certain gliadin variants that do not contain CD epitopes. Moreover, the most abundant variant found in line BW208 and addressed to the B genome contains only the p31-41 peptide but not any of the adaptive CD-response related epitopes.

The expression of prolamin genes in developing cereal grains is primarily controlled at the transcriptional level, where transcript abundance reflects the accumulation of proteins (Rahman et al., 1984; Bartels and Thompson, 1986). N fertilization has a strong influence on the quantity and quality type of prolamin proteins (Chope et al., 2014). The application of high N fertilizer increases the levels of accumulation of total, α-, γ-, and ω-gliadins in bread wheat (Chope et al., 2014; Zhen et al., 2020). So, N application strategies are one of the most common management practices to regulate yields and protein content in wheat (Nakano and Morita, 2009; Yu et al., 2018). The two RNAi lines used in this work differ in the endosperm-specific promoters used for silencing; a D-hordein promoter (Pistón et al., 2008) in the case of line D783, and a γ-gliadin promoter (Pistón et al., 2009) in line D793. These two promoters present well-differentiated expression patterns, and have different conserved N-responding regulatory elements which modulate gene expression at the transcriptional level (Forde et al., 1985). Furthermore, a comparison of the promoter sequences from A and B genomes of α-gliadin genes from bread wheat showed differences in regulatory elements (Van Herpen et al., 2008), which could explain the observed differences in the temporal expression pattern among the α-gliadin genes during the course of seed maturation (Kawaura et al., 2005). Consequently, RNAi silencing, as well as the target α-gliadin transcripts, could be affected differently by N treatments.

RT-qPCR data showed that the expression of α-gliadins was significantly increased in the wild type with the high N treatment. Both RNAi lines had consistently lower α-gliadin expression levels than the wildtype across the two N treatments, confirming the effectiveness of the silencing fragment. However, in the case of line D783, it displayed higher expression of α-gliadins transcripts when N increased, which was particularly evident at 18 DPA. In contrast, no increment in the expression level was observed for line D793, showing the high efficiency of the γ-gliadin promoter in promoting the α-gliadin silencing under different N conditions. The higher expression of the RNAi silencing fragment in line D793 might explain the higher efficiency of this line for α-gliadin silencing. In addition, differences in the temporal expression pattern between both promoters (Pistón et al., 2008, 2009) may also explain the observed differences between both RNAi lines in the silencing of the α-gliadins during grain development. The expression profile of α-gliadins in the wildtype BW208 showed a temporal expression profile similar to that described for γ-gliadin promoter (Pistón et al., 2009), with a peak at 18 DPA and then decreasing in later development stages, which may favor the higher and more stable silencing seen in line D793. In any case, differences between both RNAi lines for the silencing efficiency might due to transcriptional control, as both RNAi silencing fragments are identical. Interestingly, we did not observe a significant response in the expression of the RNAi silencing fragment for the two N levels used in this experiment, despite both D-hordein and γ-gliadin promoters contain motifs elements that were related with N response.

The sequencing of the α-gliadin variants transcriptome in this study confirms that the entire set of genes encompassing this α-gliadins amplicon are not equally expressed, and also shows differences between the wildtype and the two low-gliadin RNAi lines. In the wildtype, the transcripts abundance of pseudogenes was lower in comparison with putative genes, but interestingly, higher response to N of the pseudogenes becomes evident when the RNAi silencing mechanism is involved. A higher response to N is also observed in a group of putative genes in line D783 at 18 DPA, which confirms the RT-qPCR data described above, and may explain the higher content of α-gliadins observed in the grain for this line (García-Molina and Barro, 2017). In contrast, this increase is not observed in the same group of putative genes for line D793, indicating a better silencing in this RNAi line that is independent of N availability. Moreover, PCA data confirmed that despite the N fertilization level, line D793 was negatively associated with the expression of α-gliadin putative genes, and positively with some pseudogenes. In contrast, the higher accumulated expression of α-gliadin genes of the wildtype line correlated with a higher content of this type of prolamins with respect to the RNAi lines, regardless of the N level used. The differences between the wild type and the RNAi lines in the expression profile of the α-gliadin variants reported here are consistent with the final content of α-gliadins at the proteomic level described in García-Molina and Barro (2017).

In agreement with Kawaura et al. (2005) who reported that genes from the D genome were preferentially expressed rather than those from the A or B genome, we have also found differences in the expression of putative genes from the three wheat genomes, with the amplicon variants associated with the D genome being the most abundant. Although some studies report a lower expression of α-gliadin genes of genome B (Salentijn et al., 2009), in our study the expression of α-gliadin putative genes associated with genome B does not differ from those associated with genome A in the wildtype. Moreover, the cumulative expression of amplicon variants in the wildtype was similar for all three genomes. In contrast, N treatments increased the expression of amplicon variants from genomes A and D, which could be related to differences in the regulatory elements of the α-gliadins from different genomes (Van Herpen et al., 2008).

A comprehensive classification of α-gliadin variants was carried considering the number of epitopes as described (Ozuna et al., 2015), where only type 1 α-gliadins present CD epitopes. This classification can be useful for designing innovative RNAi or CRISPR/Cas approaches, targeting those α-gliadin types with the highest number of epitopes and that are N responsive. All α-gliadin variants classified as type 1 in this study belong to four different subtypes that showed differences in their expression. According to this classification, we found that variants containing one epitope (type 1.1-1) and six epitopes (the full 33-mer peptide, type 1.3-6) were the most affected by N treatment, increasing their expression when the N is increased. On the contrary, variants containing 2 and 4 epitopes (types 1.1-2 and 1.2-4, respectively) were less affected by N treatments. However, in the case of line D793 differences between N treatments are minimal, showing a more effective silencing, which at the end provides lower content of α-gliadins stimulatory epitopes in the grain when silencing fragment is driven by the γ-gliadin promoter (Barro et al., 2016; Sánchez-León et al., 2019).

An important concern about the reduction of gliadins is how would this affect the end use of these lines in terms of the food processing quality. In this sense, comparison among organoleptic properties of bread made with flour from different low-gliadin RNAi lines (including line D793), their wild type, and rice (as control of market-typical gluten-free bread) showed that bread made with the low-gliadin flour presented sensory and baking properties similar to those made with standard bread containing gluten (Gil-Humanes et al., 2014). Even more, the low-gliadin bread had better organoleptic properties and acceptance than rice bread, showing that they are suitable for bread-making and food processing. Regarding the nutritional value, flour from low-gliadin lines presented higher lysine content than that of their wild type, which is an essential amino acid that needs to be supplied through the diet.

It was hypothesized that the increase in N fertilization could be related to a potential increase of gluten in wheat grains and flours and thus to the spread of CD (Penuelas et al., 2020). They carried out a meta-analysis (1961–2016) and found that the increase in N fertilization rates was associated with increased content of total gluten, including all gliadin fractions, and gliadin transcripts in wheat grains, which provided a strong potential increase in the average human intake of gliadins. Some studies of populations over time showed that the prevalence of CD was increased fivefold in the United States in the period of 1975–2000 (Catassi et al., 2010). Although the causes of the increase in CD prevalence are unclear, the development of wheat varieties with a very low content of CD-related epitopes is very appealing, not only for people suffering any wheat-related pathology but also for all those who want to reduce their gluten intake. The low-gliadin D793 line, which is very effective in the silencing of gliadins, and its α-gliadin levels do not increase with increased N fertilization, could potentially be useful in reducing per capita gluten intake, particularly the highly immunogenic α-gliadins, emphasizing how biotechnology can be used to improve public health.

## Conclusion

In this work, we have used NGS technology to quantify the variants of an α-gliadin amplicon containing highly CD-stimulatory epitopes and characterized their expression in two RNAi lines with the gliadins strongly down-regulated under contrasting N treatments. In total, 45 variants have been identified, but only one contained the full complement of the 33-mer peptide. The expression of the α-gliadins amplicon is highly reduced in RNAi lines D783 and D793 in comparison to the wild type demonstrating the effectiveness of the antisense fragment. There is also a differential silencing efficiency between both RNAi lines that must be due to transcriptional control by the promoter used for silencing. High N treatment significantly increases transcripts of the α-gliadins amplicon in the wild type, and has no effect in line D793. We have also found differences in the expression of putative genes from the three wheat genomes, being those associated with the D genome the most abundant. Amplicon variants containing the full complement of 33-mer peptide (six epitopes), and those containing one epitope, were the most affected by N treatment, increasing their expression when N was increased.

The D793 RNAi line showed higher stability of down-regulation at both, protein and transcripts expression levels of α-gliadins, independently of the N treatment. This line is, therefore, of great interest to reduce gluten intake, particularly of the highly immunogenic α-gliadin variants.

## Data Availability Statement

The datasets presented in this study can be found in online repositories. The names of the repository/repositories and accession number(s) can be found in the article/Supplementary Material.

## Author Contributions

FB conceived and designed the experiments. SS-L performed the experiments. SS-L, MG, and FB analyzed the data. SS-L and FB wrote the manuscript. All authors approved the manuscript.

## Conflict of Interest

The authors declare that the research was conducted in the absence of any commercial or financial relationships that could be construed as a potential conflict of interest.

## Footnotes

1 http://www.geneious.com/
