# Supplementary material for: The α-Gliadins in Bread Wheat: Effect of Nitrogen Treatment on the Expression of the Major Celiac Disease Immunogenic Complex in Two RNAi Low-Gliadin Lines
Source: Front Plant Sci. 2021 Apr 29;12:663653. doi: 10.3389/fpls.2021.663653 (PMC8116895; doi:10.3389/fpls.2021.663653)
Supplement: Supplementary file 1 [file Presentation_1.PPTX]

## Slide 1
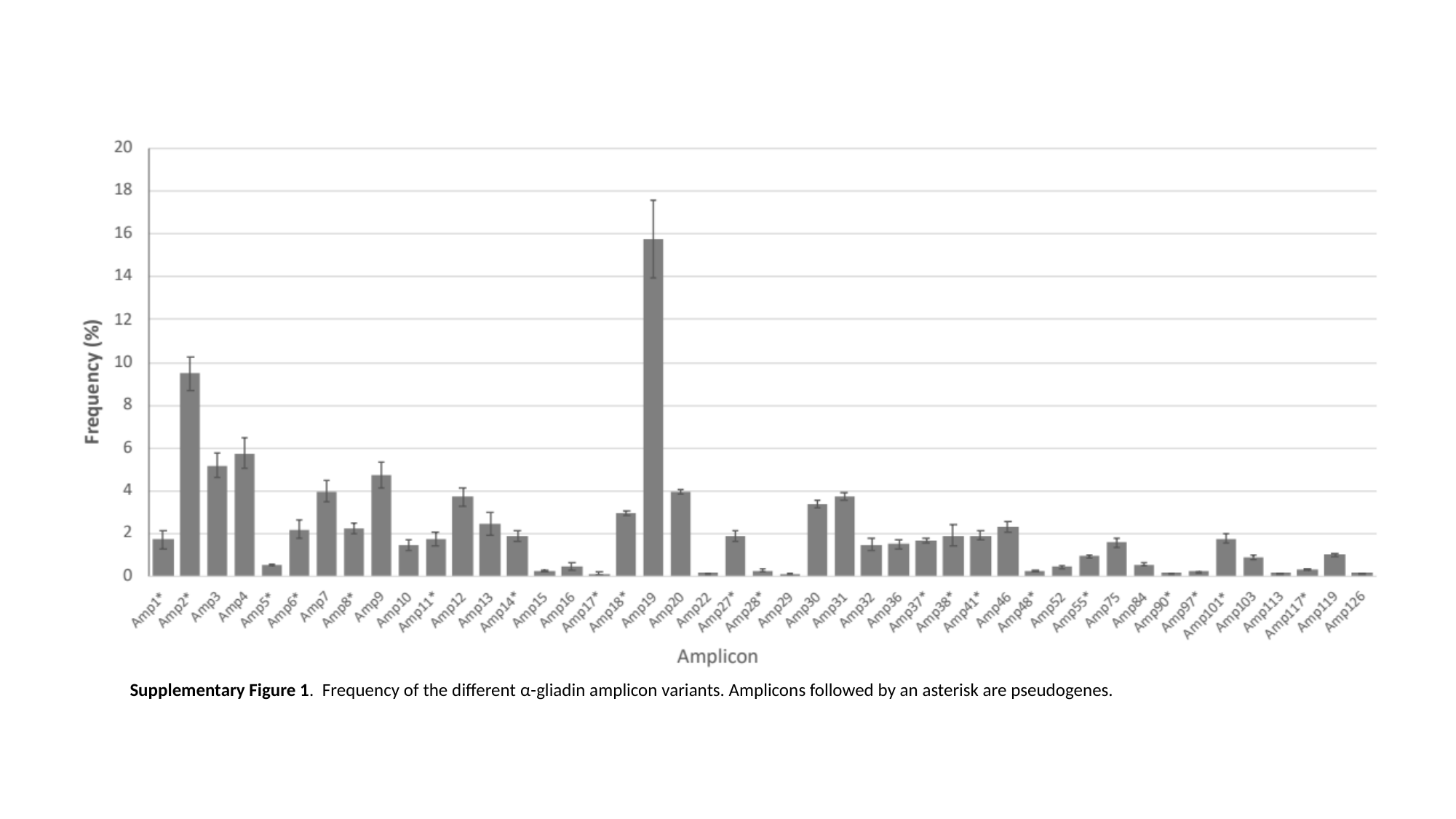

Supplementary Figure 1. Frequency of the different α-gliadin amplicon variants. Amplicons followed by an asterisk are pseudogenes.
