# Supplementary material for: The α-Gliadins in Bread Wheat: Effect of Nitrogen Treatment on the Expression of the Major Celiac Disease Immunogenic Complex in Two RNAi Low-Gliadin Lines
Source: Front Plant Sci. 2021 Apr 29;12:663653. doi: 10.3389/fpls.2021.663653 (PMC8116895; doi:10.3389/fpls.2021.663653)
Supplement: Supplementary file 2 [file Presentation_2.pptx]

## Slide 1
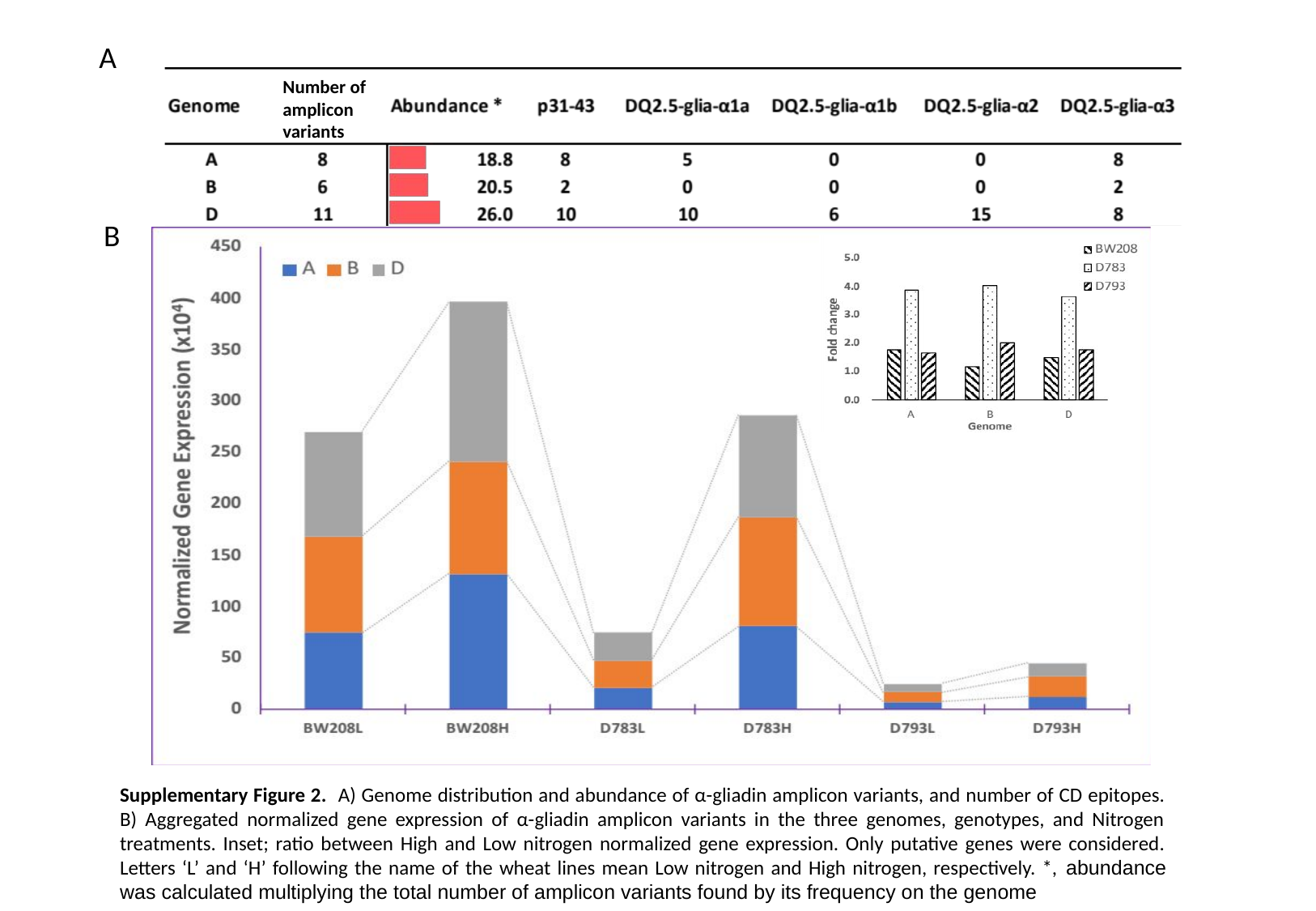

A
Number of amplicon variants
B
Supplementary Figure 2. A) Genome distribution and abundance of α-gliadin amplicon variants, and number of CD epitopes. B) Aggregated normalized gene expression of α-gliadin amplicon variants in the three genomes, genotypes, and Nitrogen treatments. Inset; ratio between High and Low nitrogen normalized gene expression. Only putative genes were considered. Letters ‘L’ and ‘H’ following the name of the wheat lines mean Low nitrogen and High nitrogen, respectively. *, abundance was calculated multiplying the total number of amplicon variants found by its frequency on the genome
